# Supplementary material for: The Epidemiology of Melioidosis and Its Association with Diabetes Mellitus: A Systematic Review and Meta-Analysis
Source: Pathogens. 2022 Jan 25;11(2):149. doi: 10.3390/pathogens11020149 (PMC8878808; doi:10.3390/pathogens11020149)
Supplement: Supplementary file 1 [file pathogens-11-00149-s001.zip › pathogens-1485727-supplementary.pdf]

Supplementary Materials

Table S1. Country wise reported melioidosis cases, 1911-2020 (Data source: Country data summary. Microbiology Department at Mahidol Oxford Tropical Medicine Re-search Unit. https://www.melioidosis.info/info.aspx?pageID=107&contentID=1070102) (4).

Country	Number of Human Case(s)	Number of Death(s)	Case Fatality Rate (%)
Thailand	27375	3378	12
Singapore	1875	126	7
Australia	1531	31	2
Cambodia	1494	193	13
Laos	1160	299	26
Malaysia	1069	20	2
India	519	24	5
Taiwan	318	0	-
Brunei	299	0	-
Sri Lanka	228	38	17
Viet Nam	166	19	11
China	91	2	2
Bangladesh*	89	20	22
Indonesia	66	12	18
Myanmar	59	2	3
Papua New Guinea	21	0	0
Hong Kong	20	2	10
New Caledonia	19	3	16
Philippines	18	2	11
Colombia	15	2	13
Brazil	14	2	14
Panama	14	5	36
Puerto Rico	10	1	10
Mexico	8	0	-
Venezuela	8	0	-
Federated States of Micronesia	7	0	-
Kenya	6	3	50
Guadeloupe	5	1	20
Madagascar	5	1	20
Aruba	3	0	-
Costa Rica	3	0	-
Seychelles	3	0	-
Trinidad and Tobago	3	1	33
Cameroon	2	0	-
Gambia	2	0	-
Guam	2	0	-
Guatemala	2	0	-
Honduras	2	0	-
Martinique	2	0	-
Pakistan	2	0	-
British Virgin Islands	1	0	-
Burkina Faso	1	0	-
Dominican Republic	1	0	-
Ecuador	1	0	-

Fiji	1	0	-
France	1	0	-
Gabon	1	1	100
Iran	1	0	-
Israel	1	0	-
Ivory Coast	1	0	-
Malawi	1	0	-
Mauritius	1	0	-
Nepal	1	1	100
Nigeria	1	0	-
Sierra Leone	1	0	-
Turkey	1	1	-
**Total**	**36514**	**4177**	**11%**

* Some cases have not been reported yet to Melioidosis.info platform (source: personal communication).

Table S2. Country with reported melioidosis cases having diabetes mellitus.

Country.	Authors	Publication Year	Number of Melioidosis Cases	Number of Melioidosis Cases Having Diabetes	Proportion of Melioidosis Cases Having Diabetes (%)	95% CI
Australia	Currie BJ et al.[40]	2021	1148	513	45	42-48
	Stewart JD et al. [111]	2017	183	103	56	49-64
	Hanson J et al.[112]	2021	321	169	53	47-58
	Gassiep I et al.[72]	2021	128	59	46	37-55
Bangladesh	Chowdhury FR et al.[45]	2018	48	40	83	70-93
	Barai L et al.[51]	2014	19	17	89	67-99
	Afroze SR et al. [52]	2017	11	11	100	72-100
Brunei	Pande K et al. [113]	2018	115	86	75	66-82
Cambodia	Rammaert B et al.[89]	2011	39	7	18	7-34
	Vlieghe E et al.[13]	2011	58	34	59	45-71
China	Fang Y et al.[16]	2015	170	74	44	36-51
	Zheng X et al.[12]	2019	277	131	47	41-53
Hong Kong	Lui G et al.[114]	2018	14	6	43	18-71
India	Koshy M et al.[42]	2019	114	93	82	73-88
	Jesudason MV et al.[57]	2003	28	8	29	13-49
	Saravu K et al.[47]	2010	25	17	68	46-85
	Vidyalakshmi K et al.[10]	2007	25	19	76	55-91
	Vidyalakshmi K et al.[95]	2012	95	72	76	66-84
	Ganesan V et al.[44]	2021	35	31	89	73-97
Laos	Rachlin A et al.[71]	2016	9	6	67	30-93
	Bulterys PL et al.[32]	2018	840	330	39	36-43
Malaysia	Puthucheary SD et al.[60]	1992	50	19	38	25-53
	Hassan MR et al.[22]	2010	145	82	57	48-65
	Mardhiah K et al.[115]	2021	453	316	70	65-74
Myanmar	Win MM et al.[92]	2021	21	5	24	8-47
Singapore	Pang L et al.[21]	2018	614	348	57	53-61
	Chan KPW et al. [39]	2005	27	16	59	39-78
	Heng BH et al.[38]	1998	372	214	58	52-63
	Lo TJ et al.[11]	2009	693	332	48	44-52
Sri Lanka	Corea EM et al.[116]		250	163	65	59-71
Taiwan	Ko W et al. [14]	2007	40	20	50	34-66

	Chou D et al.[48]	2007	30	15	50	31-69
Thailand	Churuangsuk C et al.[19]	2016	134	63	47	38-56
	Reechaipichitkul W [20]	2004	162	74	46	38-54
	Waiwarawooth J et al.[65]	2008	83	48	58	46-69
	Limmathurotsakul D et al. [73]	2006	921	524	57	54-60
	Limmathurotsakul D et al.[30]	2010	2243	662	30	28-31
	Chaichana P et al.[82]	2021	194	112	58	50-65
Vietnam	Phuong DM et al.[117]	2008	40	10	25	13-41

Table S3. Association between diabetes mellitus and melioidosis.

**Country**	**Authors**	**Publication Year**	**Odds Ratio (OR)**	**Relative Risk (RR)**	**95% CI**
Australia	Pitman MC et al. [118]	2015	5.63	-	1.12-54.41
Australia	Currie BJ et al. [31]	2010	1.5	-	1.04-2.1
Thailand	Suputtamongkol Y et al. [88]	1999	5.9	-	4.0-8.9
Australia	Currie BJ et al. [90]	2004	-	13.1	9.4-18.1
Australia	Merianos A et al. [94]	1993	-	12.9	5.1-32.7
Thailand	Limmathurotsakul D et al. [30]	2010	-	12.4	11.2-13.7
